# Prevalence, median time, and associated factors with the likelihood of initial antidepressant change: a cross-sectional study in Qatar

**DOI:** 10.1186/s12888-021-03099-0

**Published:** 2021-02-22

**Authors:** Nervana Elbakary, Sami Ouanes, Sadaf Riaz, Oraib Abdallah, Islam Mahran, Noriya Al-Khuzaei, Yassin Eltorki

**Affiliations:** 1 Department of Pharmacy, Mental Health Services, Hamad Medical Corporation, Doha, Qatar; 2 Medical Department, Mental Health Services Hamad Medical Corporation, Doha, Qatar

**Keywords:** Initial antidepressant, Dose optimization, Major depressive disorder, Comorbid anxiety, Combination, Augmentation, Switching, Premature discontinuation

## Abstract

**Background:**

Major Depressive Disorder (MDD) requires therapeutic interventions during the initial month after being diagnosed for better disease outcomes. International guidelines recommend a duration of 4–12 weeks for an initial antidepressant (IAD) trial at an optimized dose to get a response. If depressive symptoms persist after this duration, guidelines recommend switching, augmenting, or combining strategies as the next step. Premature discontinuation of IAD due to ineffectiveness can cause unfavorable consequences. We aimed to determine the prevalence and the patterns of strategies applied after an IAD was changed because of a suboptimal response as a primary outcome. Secondary outcomes included the median survival time on IAD before any change; and the predictors that were associated with IAD change.

**Methods:**

This was a retrospective study conducted in Mental Health Services in Qatar. A dataset between January 1, 2018, and December 31, 2019, was extracted from the electronic health records. Inclusion and exclusion criteria were defined and applied. The sample size was calculated to be at least 379 patients. Descriptive statistics were reported as frequencies and percentages, in addition, to mean and standard deviation. The median time of IAD to any change strategy was calculated using survival analysis. Associated predictors were examined using several cox regression models.

**Results:**

A total of 487 patients met the inclusion criteria of the study, 431 (88%) of them had an occurrence of IAD change to any strategy before end of the study. Almost half of the sample (212 (49%); 95% CI [44–53%]) had their IAD changed less than or equal to 30 days. The median time to IAD change was 43 days with 95% CI [33.2–52.7]. The factors statistically associated with higher hazard of IAD change were: younger age, un-optimization of the IAD dose before any change, and comorbid anxiety.

**Conclusions:**

Because almost half of the patients in this study changed their IAD as early as within the first month, efforts to avoid treatment failure are needed to ensure patient-treatment targets are met. Our findings offered some clues to help clinicians identify the high-risk predictors of short survival and subsequent failure of IAD.

## Background

Major depressive disorder (MDD) is considered a severe and often recurrent medical illness that most often restricts the normal functioning of life. It is highly prevalent worldwide and is associated with a significant negative impact on productivity and quality of life [1]. An estimate of 13.5% of adults in Qatar has had at least one major depressive episode during their life [2]. It is preferable to seek medical and pharmacotherapeutic care during the initial month of being diagnosed in order to achieve remission [3].

The first-line treatment for MDD can consist of psychotherapy and/or antidepressant (AD) medication [4]. A combination of both psychotherapy and pharmacotherapy might be necessary in some cases, depending on the severity of the illness and the patient’s treatment preference [4–6]. If depressive symptoms persist after an adequate trial (4–12 weeks) of the initial antidepressant (IAD) at an optimized dose, clinical practice guidelines recommend switching to an alternate selective serotonin reuptake inhibitor (SSRI), serotonin-norepinephrine reuptake inhibitor (SNRI), bupropion or mirtazapine [4]. Alternatively, it is possible to either combine the IAD with an AD that has an alternative mechanism of action or to augment the IAD with a second-generation antipsychotic, lithium or psychotherapy [7, 8]. Generally, if there is less than 50% improvement in a depression psychometric scale after 12 weeks of an optimized dose of IAD, switching to a different AD should be strongly considered. If a response to therapy (50% reduction in symptoms in a depression psychometric scale) has occurred within 6 weeks of an adequate trial, the AD should be continued at an optimal dose and re-evaluated at 8 and 12 weeks [9].

However, despite a variety of currently available treatment options, many patients do not respond early enough in the course of a major depressive episode. Even among patients who show some response, residual symptoms often persist and can impact the patient’s quality of life [7]. This was indicated by a study done on outpatients with MDD which showed that even after receiving an adequate trial of a first-line treatment such as an SSRI, only 29–46% of patients showed an adequate response [10]. Similarly, a large multicenter study showed that only a few patients with MDD were able to attain remission within a period of 10 to 14 weeks [11].

Before the patient is “labeled” treatment-resistant, an attempt to optimize the IAD trial is essential by ensuring that the maximum recommended dose for the recommended duration has been used. A trial duration of approximately 4 to 6 weeks is recommended by most of the guidelines for the treatment of depression [12, 13]. Results from the sequenced treatment alternatives to relieve depression (STAR*D) trial indicated that a longer trial period may be required for treated patients to develop the full therapeutic potential of the intervention. For example, of all participants who eventually remitted using the IAD, up to one half did so between 6 and 12 weeks [14, 15]. Nevertheless, early signs of response that can be observed with early improvement (often defined by at least 20% reduction in a depression scale) at two weeks are capable of predicting remission state at the end of 12 weeks [16].

Most patients with MDD in the mental health setting have been labeled incorrectly as treatment-resistant where in fact they have not been subjected to an adequate trial of guideline-recommended therapy [14]. Even with the eventual recovery, some patients might need a trial-and-error strategy, as there is currently no clear way to predict the response of a particular patient to a particular drug, and many may become unresponsive to AD treatments by the time [17]. Careful step-by-step placement of AD medications at optimum doses and duration, with frequent evaluation of efficacy, can help prevent the disease from progressing to a treatment-resistant phase. Avoiding irrational practices such as subtherapeutic doses of IAD, premature switching between the ADs, and refraining from unjustified polypharmacy can help the disease to go into a remission phase [18]**.**

In this study, we were interested in finding out the prevalence and the patterns of strategies applied after an IAD changed because of a suboptimal response towards alleviating the depressive symptoms as a primary outcome. Additionally, the secondary outcomes studied were the frequency and percentages of different change strategies applied to the IAD after suboptimal response at different time points; the median survival time on IAD before any change; and the possible risk or protective factors that were associated with the likelihood of IAD change.

## Methodology

### Study design, setting, and participants

This was a retrospective study conducted in the Mental Health Services (MHS), Hamad Medical Corporation (HMC) in Qatar. A dataset was extracted from the electronic health records (EHR) system (Cerner system®).

Participants were eligible for inclusion in the study if they: (i) were patients who visited the Mental Health Service as an outpatient or as an inpatient, and if they were diagnosed with MDD according to the Diagnostic and Statistical Manual of Mental Disorders, Fifth Edition (DSM-5) criteria [19]. (ii) had a new MDD episode, whether first episode, a relapse, or a recurrence. It was difficult in a clinical practice setting to differentiate between relapse and recurrence; hence, we included both groups into a relapse (or retreatment) group for convenience. (iii) started treatment with a single AD (using a flexible-dose regimen if required). (iv) had an age of 18 to 70 years old on their index date (the first date of prescribing the IAD) (v) had an index date between January 1, 2018, and December 31, 2019. Our follow-up period took place from an index date of each patient to the date of IAD change to any strategy (switching, augmentation, or combination).

Patients were excluded if they: (i) had a prescription of AD within 6 months or less prior to the index date of initial diagnosis as MDD. (ii) had a psychotic disorder or an MDD with psychotic features, in whom antipsychotic treatment was initially prescribed, along with the AD, (iii) had a treatment-resistant depression for whom multiple MDD treatments were deemed necessary when used simultaneously in their index date (iv) had bipolar depression, obsessive-compulsive disorder, or post-traumatic stress disorder in whom giving treatment with AD was warranted. (v) Patients who were pregnant MDD on their index date. (vi) Treatment with electroconvulsive therapy, transcranial magnetic stimulation, monoamine oxidase inhibitors.

### Sample size and sampling techniques

Based on a pilot study we did in the same setting one year earlier on 70 patients with MDD, 40% of the sample had a change to their IAD within less than 28 days. Thus, with an estimate for a proportion of 0.4 and a margin of error of 0.05, the needed sample size to achieve power was at least 379 patients or more. 95% confidence interval was used. Two-sided *p*-value was considered significant if it was less than 0.05. The extracted data were validated, de-identified, and listed in excel sheets by six healthcare professionals including the pharmacy informatics specialist. Typical purposive sampling was applied by reviewing 800 patient charts for eligibility consideration.

### Variables and statistical analysis

The identified IAD with their frequencies/ percentages were tabulated. Index date from IAD to the nearest one of three pre-planned change strategies was calculated. The first change strategy was switching which was defined as discontinuation of the IAD and administration of another AD. The second change strategy was augmentation which was defined as the addition of another drug, that is not an AD, to the IAD; and it was pre-determined by the team members to dedicate this category only for antipsychotic use. The third change strategy was combination strategy which was defined as either a) switch from IAD to another AD with the addition of another drug, which is an AD or, b) combined administration of IAD with another AD.

Descriptive statistics were reported as relative frequencies in percentages as well as mean ± SD/median ± IQR (interquartile range). The Shapiro-Wilk test was used to test for normality. Median survival time of IAD before any change strategy was plotted using the Kaplan Meier curve. Censored patients were either lost to follow-up or had no occurrence of IAD change prior to the end of the study. Time (in days) from the IAD index date to any change strategy or censoring was calculated and labeled as survival time on IAD.

The association between the risk of IAD change and only seven independent variables was studied to avoid overfitting of the model. The independent variables, namely age, gender, bothersome side effects, substance use, un-optimization of the IAD dose before any change, comorbid anxiety, and first experience MDD episode were chosen based on literature review and clinical judgment of experts in our hospital. All the independent variables used were categorical except for age. The IAD dose was considered optimized if was equal or more than fluoxetine 40 mg or venlafaxine 150 mg equivalent doses per day [8].

A univariate cox proportional hazard model used first to study the association between the risk of IAD change and individual factors to report crude hazard ratio with 95% confidence interval. Then these factors were entered in two subsequent multivariate cox proportional hazard models to get the adjusted hazard ratio (AHR) with a 95% confidence interval. The multivariate regression was done initially using a complete case analysis (CCA) approach. Then a final multivariate regression model was performed using multiple imputations (MI) to replace all the missing values in each independent variable. For sensitivity analysis, we used all the available data in the MI to create and analyze 20 multiply imputed models for each independent variable with missing data. Data analysis was performed using IBM SPSS Statistics for Windows (Version 25.0).

### Ethics

This study was conducted in accordance with the Declaration of Helsinki and the Qatari ethical guidelines for medical and health research involving human subjects. Prior to the initiation of the study, the study protocol was reviewed and approved by the institutional review board (IRB) of the Medical Research Center (MRC) in HMC under protocol number (MRC-01-20-055). Since this was a retrospective chart review, it was exempted by MRC from the requirement of informed consent. The administrative permissions were attained by the research team to access the data used in the research.

## Results

A total of 487 patients met the inclusion criteria of the study. Only 431 (88%) had their IAD changed to any change strategy before the end of the study and 57 (12%) patients were considered as censored.

Table 1 represents the demographics and patient characteristics of 487 patients. The average age for participants was 39.1 ± 12.3 years, with 204 (41.9%) out of them aged 18–34 years old while 174 (35.7%) aged from 35 to 49 years old. For ethnicity, Arabs either from East Mediterranean / North Africa 186 (38.2%) or from the Gulf area 160 (32.9%) were more prevalent than others. Males were slightly higher than females 54.8 and 45.2% respectively. Patients with first experience MDD episode 255 (52%) constituted a major part of our sample comparing to the relapse group 206(42%) which could be explained in the context of our restricted inclusion and exclusion criteria. The majority of our patients had their IAD from the SSRI group versus other non-SSRI groups 390 (80%) vs 97 (20%) respectively. SSRIs included patients who started on escitalopram (43%), fluoxetine (20%), fluvoxamine (0.2%), paroxetine (4%), sertraline (12%) as their IAD, while non-SSRIs included Duloxetine (5%), Venlafaxine (6%), and Agomelatine (0.1%).

**Table 1:** Demographics and patient characteristics

Parameter	*N* (%)
**Age group**	
18–34	204 (41.9)
35–49	174 (35.7)
50–64	101 (20.7)
≥ 65	8 (1.6)
**Age in years**	(mean, SD): 39.09 ± 12.3; (Median, IQR): 37 ± 18
**Gender**	
Male	267 (54.8)
Female	220 (45.2)
**Ethnicity groups**	
Arab/ Gulf	160 (32.9)
Arabs/East Mediterranean& North African	186 (38.2)
Asian	99 (20.3)
White	27 (5.5)
Black African	15 (3.1)
**Marital Status**	
Married	181 (37)
Single	92 (19)
Unknown	214 (44)
**Type of episode**	
First experience of MDD episode	255 (52)
Relapse	206 (42)
Missing	26 (5)
**IAD**	
Duloxetine	25 (5)
Escitalopram	211 (43)
Fluoxetine	98 (20)
Fluvoxamine	1 (0.2)
Mirtazapine	38 (7)
Paroxetine	20 (4)
Sertraline	60 (12)
Venlafaxine	29 (6)
Agomelatine	5 (0.1)

*N* number of patients included

*IAD* Initial Antidepressant

*MDD* Major Depressive Disorder

Table 2 represents cumulative and absolute prevalence of the three IAD change strategies (switching, augmentation, and combination) at different time points. A total of 431 patients were included in the analysis. This number represented the patients who showed occurrence of any change to their IAD due to suboptimal response before the end of the study on December 31, 2019. Almost half of the sample (212 (49%); 95% CI [44–53%]) had their IAD changed within less than or equal to 30 days. The absolute prevalence of IAD change that occurred in 30 days or less was significantly higher than changing the IAD within 90 days (87 (20%); 95% CI [17–24%]), within 180 days (83 (19%); 95% CI [15–23%]), or after more than 180 days (49 (11%); 95% CI [8–14%]).

**Table 2:** Cumulative and absolute prevalence of the three IAD change strategies (switching, augmentation, and combination) at different time points (*N* = 431) ^#^

Prevalence of IAD change at different time points	Cumulative prevalence (All strategies)	Absolute prevalence (All strategies)	Detailed cumulative prevalence expressed as frequency, percentages & 95% CI of each IAD change strategy		
			Switching	Augmentation	Combination
≤ 30 days	212 (49%) [44–53%]	212 (49%) [44–53%]	144 (68%) [61–74%]	25 (12%) [7.4–16%]	43 (20%) [15–25%]
≤ 90 days	299 (69%) [65–74%]	87 (20.2%) [17–24%]	207 (69%) [64–74%]	32 (11%) [7–14%]	60 (20%) [15–25%]
≤ 180 days	382 (88%) [85–91%]	83 (19.2%) [15–23%]	259 (68%) [63–72%]	41 (11%) [8–14%]	82 (21%) [17–26%]
≥ 181 days *	431 (100%)	49 (11.4) [8–14%]	294 (68%) [64–73%]	46 (11%) [7–14%]	91 (21%) [17–25%]

*IAD* Initial Antidepressant, *CI* Confidence Interval, ^#^ Number of patients with occurrence of IAD changes (i.e.: excluding censored patients); *to end of study

Among the different IAD change strategies, switching was consistently more common than combination or augmentation, within 30, 90, or 180 days, as well as more than 181 days. The total cumulative number of patients for whom switching was carried out during the observation time of the study was 294 out of 431 (68, 95% CI [64–73%]). Overall, the frequency of patients who were switched from any IAD to SSRIs was 118 (40%); 95% CI [34–46%], from any IAD to an atypical AD (mirtazapine, bupropion, agomelatine, and vortioxetine) were 108 (37%); 95% CI [31–43%], from any IAD to SNRIs were 41 patients (14%); 95% CI [10–18%] and lastly, from any IAD to tricyclic ADs were 27 (9%); 95% CI [6–13%].

**Fig. 1:**
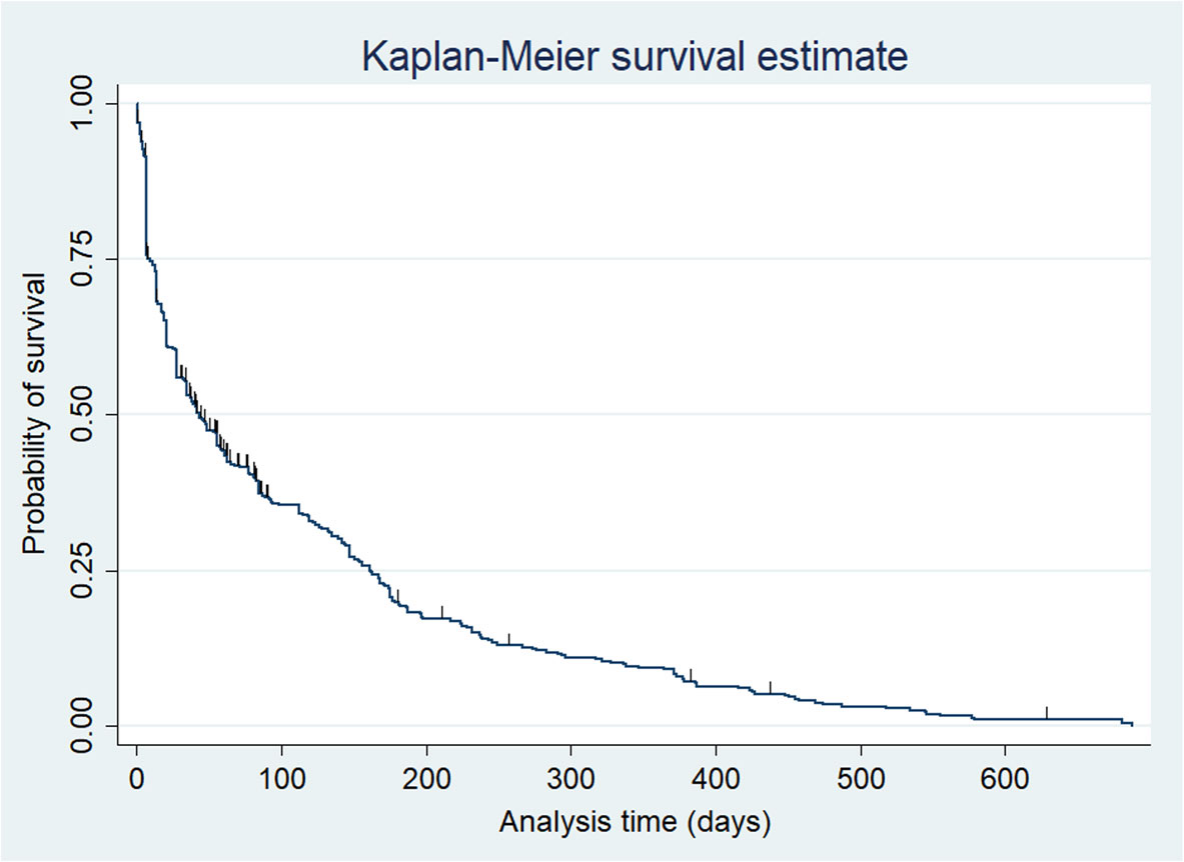
Kaplan- Meier Survival Estimate

Following the switching strategy, the combination strategy was the second most common strategy with a total of 91 out of 431 (21, 95% CI (17–25%). Lastly, augmentation of IAD with antipsychotics had the lowest prevalence of 46 out of 431 (11, 95% CI 7–14%). Of all augmentation strategies, the occurrence of augmentation with second-generation antipsychotics (SGAs) was higher (*n* = 33, 72, 95% CI 57–84%) than augmenting with first-generation antipsychotics (FGAs) (*n* = 13, 28, 95% CI 16–43%). Among SGAs, quetiapine was the most common add-on therapy used in 18 cases (55, 95% CI 36–72%). Median time to IAD change or the survival time of patients on the first IAD before any change strategy was 43 days with 95% CI [33.2–52.7]. Kaplan Meier survival estimate and summary statistics about survival time are shown in Fig. 1 and Table 3.

**Table 3:** Summary statistics about survival probability on IAD and time to IAD change

Time at risk (in days)	Incidence rate of IAD change (Person- days)	No. of subjects included in survival analysis	Survival probability on IAD& time to IAD change (in days)			
			Probability	25%	50%	75%
44,941.9	0 .0095902	487	Time	9	43	162

*IAD* Initial Antidepressant

As shown in Table 4, five independent variables (age, bothersome side effects, un-optimization of the dose before any change, comorbid anxiety, first onset episode) were significantly associated with the likelihood of IAD change in the crude analysis. However, these crude results changed after the simple cox regression model was extended to multiple cox regression. CCA multivariate regression model showed two statistically significant predictors: un-optimization of the dose before any change, and co-morbid anxiety. The final MI cox regression model showed three statistically significant predictors. First, patients with unoptimized IAD doses had a 35% higher hazard of IAD change compared to those with optimized doses (AHR 1.35, 95% CI [1.046–1.745]; *P*-value = 0.022) after controlling for other variables in the model. In other words, results could be expressed as a 26% decrease in the survival time on IAD was occurred as a result of un-optimization of the IAD dose (26% as 1/1.35 = 0.74). Second, patients without co-morbid anxiety had a 24% less hazard of IAD change as compared to those with co-morbid anxiety (AHR 0.756, 95% CI [0.617–0.926]; *P*-value = 0.007) after controlling for other variables in the model; or a 32% prolongation of the survival time on IAD was occurred due to absence of comorbid anxiety (32% as 1/0.756 = 1.32). Third, age was statistically significant with a 1.2% decrease in the hazard of IAD change for every year increase in the patient’s age (AHR 0.988, 95% CI [0.98–0.99], *P*-value = 0.006) adjusted for other variables in the model. In this context, older patients (by ten years) had a 12% prolongation in the length of survival time on IAD when compared to younger patients (1/0.988 = 1.012). Other studied variables (gender, bothersome side effects, substance use, first experience MDD episode) were not statistically associated with the hazard of IAD change or the survival time on IAD in the final MI model.

**Table 4:** Cox proportional hazard regression with risk and protective predictors for IAD change

Risk or protective predictors	Sub-group	***n*** (%)	Reference for categoric-al variables	Univariate cox proportional hazard regression (***n*** = 487)			Multivariate cox proportional hazard regression					
							Complete case analysis (CCA) (***n*** = 277)			After Multiple imputation (MI) (***n*** = 487)		
				Crude HR	95% C. I	***P***-value	AHR	95% C. I	***P***-value	AHR	95% C. I	***P***-value
**Age (Years)**				0.987	0.985–0.995	0.001**	0.997	0.986–1.008	0.59	0.988	0.980–0.997	0.006**
**Gender**	Male	220 (45.2%)		0.909	0.751–1.10	0.327	0.945	0.737–1.21	0.66	0.862	0.708–1.05	0.14
	Female	267 (54.8%)	1									
**Bothersome side effects**	No	331 (83.8%)		0.713	0.541–0.938	0.016**	1.09	0.79–1.51	0.60	1.256	0.967–1.632	0.087
	Yes	64 (16.2%)	1									
**Substance use**	No	337 (87.5%)		0.806	0.556–1.167	0.253	0.836	0.54–1.29	0.42	0.781	0.560–1.089	0.145
	Yes	48 (12.5%)	1									
**Un- optimization of IAD dose before any change**	Un-optimized	277 (56.3%)		1.446	1.18–1.77	0.000**	1.36	1.06–1.76	0.02**	1.35	1.046–1.745	0.022**
	Optimized	147 (34.7%)	1									
**Comorbid anxiety**	Absent	207 (44.6%)		1.42	1.165–1.732	0.001**	0.69	0.54–0.88	0.003**	0.756	0.617–0.926	0.007**
	Present	257 (55.4%)	1									
**First experience episode**	No	205 (44.6%)		1.273	1.044–1.552	0.017**	1.16	0.904–1.48	0.24	1.188	0.966–1.460	0.102
	Yes	255 (55.4%)	1									

** statistically significant; *AHR* adjusted hazard ratio, *IAD* initial antidepressant

## Discussion

To our knowledge, this is the first study to assess the prevalence and patterns of strategies applied after an IAD changed because of a suboptimal response in patients with MDD in Qatar and the Middle East.

In this retrospective study, we found that the IAD was an SSRIs in 80% of the cases. Almost half of the patients (49% [44–53%]) had their IAD changed within less than one month, whereas the proportions of patients whose IAD changed in 31 to 90 days, 91 to 180 days, or more than 180 days were lower (20, 19, and 11% respectively). The most common IAD change strategy used was switching, mostly to an SSRI or to an atypical AD (40 and 37% respectively), followed by combination strategy (21%), then augmentation with antipsychotics (11%). The median time to IAD change was 43 days [33.2–52.7]. The factors which were statistically associated with higher hazard of IAD change: younger age, un-optimization of the IAD dose before any change, and comorbid anxiety.

ADs are indicated for the treatment of depression, generalized anxiety disorders, obsessive-compulsive disorder, and post-traumatic stress disorder. There are 13 ADs in HMC formulary representing the different AD classes. ADs take considerable time to induce either response or remission and for many patients, the response is considered suboptimal [7]. This lag in the AD response may lead to negative MDD outcomes including increased risk of suicidal behavior and other deliberate self-harm, psychological distress, occupational and functional limitations, and lack of adherence to medications. The results of this study showed that almost half of the patients underwent a change in their IAD treatment due to a perceived lack of response in the first 30 days of the treatment. Our median time to IAD change of 43 days was very similar to the one reported in a previous UK study (44 days) [20]. However, the change of the IAD strategy seemed to be much slower in a French study where only 16% had their initial AD switched over a 90-day window [21]. Similarly, in American study where only 8.6% of participants had their initial AD switched and only an additional 2.4% had a second AD combined with the first one, during the 90 first days of AD use [22]. In an Italian study, the proportion of patients switched to a second AD over one year of follow-up was as low as 0.7% [23]. These differences between studies can be explained by different populations (primary versus secondary or tertiary care), as well as the differences in available resources and local or national guidelines. The International guidelines such as National Institute for Health and Care Excellence (NICE-2018) and the Canadian Network for Mood and Anxiety Treatments (CANMAT-2016) generally specify a timeframe of 2–4 weeks to switch from the IAD at an adequate dose if no response was observed [6].

We found that a quicker change in the IAD strategy was associated with younger age. There has been some evidence that older patients might exhibit a slightly slower response to AD medication even though other studies did not show any link between age and speed of response [20, 24]. The possibly slower response in older patients might explain why the time to change IAD in our study was longer in older individuals. In addition, since polypharmacy is much more common in older patients, it is understandable that clinicians could be more reluctant to prescribe yet another drug, and it seems wise to wait longer before having to combine ADs or augment the AD medication with an antipsychotic drug [25, 26]. This goes in line with international guidelines recommending monotherapy in older patients with MDD [4].

Patients with unoptimized IAD doses also had a higher hazard to undergo a change in their IAD. This might be due to some clinicians resorted to switch, combine, or augment IAD without optimizing the IAD dose first. Since we basically relied on documentation of the physicians to report the reasons behind IAD change, we found some patients’ files with unclear justifications whether those patients underwent a change to their IAD due to side effects or suboptimal response. To reduce bias or imprecision, we decided to perform adjustment for “bothersome side effects” variable in the list of independent factors included in the regression models. However, the final MI multivariate model showed non-statistically significant association with the likelihood of IAD change.

Even though the conventional approach remains to optimize the dose of the AD medication before switching or adding another psychotropic medication, there has been some evidence that “early switching strategies” might actually yield better MDD outcomes [16, 27, 28]. A meta-analysis showed that the lack of early improvement (often defined by less than 20% reduction in a depression scale score) at two weeks may indicate that changes in depression management should be considered earlier than conventionally thought [16]. Meanwhile, a double-blind, randomized study showed that the time to regaining normal functionality might be shorter when adopting the early compared to the conventional switching strategy [27]. Another recent meta-analysis of nine studies showed significant associations between early improvement, response, and remission. Nevertheless, the treatment scenario associated with the best remission rate was switching at four weeks rather than at two or six weeks [29].

Changing the IAD strategy was slower in patients without comorbid anxiety. This was consistent with previous studies showing that patients with anxiety symptoms concurrently with the depressive illness might have a slower onset of action of AD medication [20]. Similarly, in level one of STAR*D, MDD patients with anxiety symptoms exhibited a significantly slower remission and poorer outcomes than patients without comorbid anxiety [30]. Hence, in our study it seems clinically understandable for patients without comorbid anxiety to survive longer and show better response on their IAD.

The findings of this study can have direct clinical guidance for health care professionals since an optimized, evidence-based use of AD medication can improve the clinical outcomes of patients with MDD; and also, to identify high-risk factors that could worsen the survival time on IAD such as young age and comorbid anxiety [31]. We included most of the presently available AD medications commonly used in a clinical practice setting, and we compared median IAD change time and percentages in an actual clinical situation in both inpatient and outpatient settings.

Despite the merits, several limitations in our study need to be acknowledged: the retrospective design and the reliance on medical records led to missing data. Nevertheless, most evidence comes from retrospective studies (with their inherent limitations), or from trials where “the most complex” patients are excluded (including patients with multiple comorbidities, with a neurocognitive disorder, or with acute suicidality). This may partly explain the gap that exists between the data of the literature and the clinical practice. In addition, we did not include the “IAD pharmacological group” variable in the Cox regression model because the number of patients who had their IAD as SSRIs was incomparable to those on non-SSRIs. Our sample was also recruited from the main psychiatric hospital in the state; however, patients with MDD treated in primary healthcare centers or in the private sector might have different severity of illness; hence, our findings can probably be extrapolated to the entire population of patients treated with AD medications with caution. Despite having more clinical significance with dichotomizing the variable of dose optimization as optimized and non-optimized, authors believed that pooling the standardized means of IAD doses as a continuous variable in mg/day could have been a better approach to avoid loss of more information about the dose relationship with survival on IAD. Furthermore, the use of psychometric scales to assess the efficacy of the AD medication could have provided a much more objective and quantifiable assessment of the response; however, in this setting, measurement-based care (MBC) is not being routinely used. Finally, adherence was not assessed, and it was possible that adherence problems could have resulted from other residual confounders.

## Conclusion

To date, a consensus still does not exist as to how long patients should stay on their IAD before it is deemed ineffective. Our study investigated in a real setting the median survival time on IAD before any change due to suboptimal response. Because almost half of the patients changed their IAD as early as within the first month, efforts to avoid treatment failure are needed to ensure patient-treatment targets are met. Findings from this study offered some clues to help clinicians identify the high-risk predictors of short survival and subsequent failure of IAD such as: younger age, un-optimization of the dose before any change, and comorbid anxiety. Future research should be directed at determining the factors leading patients to change their IAD in prospective studies with adequate randomization powered enough to reliably study those factors as a primary outcome.

## Data Availability

The datasets used and analyzed during the current study are available from the corresponding author upon a reasonable request.

## Abbreviations

AD: Antidepressant
AHR: Adjusted hazard ratio
CANMAT: Canadian Network for Mood and Anxiety Treatments
CCA: Complete Case Analysis
CI: Confidence Interval
DSM-5: Diagnostic and Statistical Manual of Mental Disorders, Fifth Edition
EHR: Electronic Health Record
FGA: First-generation antipsychotic
HMC: Hamad Medical Corporation
IAD: Initial antidepressant
IQR: Interquartile range
MBC: Measurement-based care
MDD: Major Depressive Disorder
MHS: Mental Health Services
MI: Multiple imputation
NICE: National Institute for Health and Care Excellence
SGA: Second-generation antipsychotic
SNRI: Serotonin-norepinephrine reuptake inhibitor
SSRI: Selective serotonin reuptake inhibitor
STAR*D: Sequenced Treatment Alternatives to Relieve Depression
